# Free radical scavenging capacity, anticandicidal effect of bioactive compounds from *Sida Cordifolia* L., in combination with nystatin and clotrimazole and their effect on specific immune response in rats

**DOI:** 10.1186/1476-0711-11-33

**Published:** 2012-12-26

**Authors:** Maurice Ouédraogo, Kiessoun Konaté, Alexis Nicaise Lepengué, Alain Souza, Bertrand M’Batchi, Laya L Sawadogo

**Affiliations:** 1 Laboratory of Animal Physiology, University of Ouagadougou, 09 P.O. Box: 848, Ouagadougou, 09, Burkina Faso; 2 Laboratory of Biochemistry and Applied Chemistry, University of Ouagadougou, 09 P.O. Box: 848, Ouagadougou, 09, Burkina Faso; 3 Laboratory of Plant Pathology, Faculty of Science, University of Science and Technology of Masuku, Franceville, P.O. Box: 943, Franceville, Gabon; 4 Laboratory of Animal Physiology, Electrophysiology and Pharmacology, Faculty of Science, University of Science and Technology of Masuku, P.O. Box: 943, Franceville, Gabon

## Abstract

**Background:**

Infectious diseases caused by fungi are still a major threat to public health, despite numerous efforts by researchers. Use of ethnopharmacological knowledge is one attractive way to reduce empiricism and enhance the probability of success in new drug-finding efforts. In this work, the total alkaloid compounds (AC) from *Sida cordifolia* L. (Malvaceae) have been investigated for their free radical scavenging capacity, antifungal and immunostimulatory properties.

**Method:**

The antifungal activity was investigated against five candida strains using the microplate dilution method and the Fractional Inhibitory Concentration Index (FICI) of compounds was evaluated. The antioxidant activity of the samples was evaluate using three separate methods, at last, the immunostimulatory effect on immunosuppressed wistar rats was performed.

**Results:**

As for the antifungal activity, result varied according to microorganism. The results obtained in this antifungal activity were interesting and indicated a synergistic effect between alkaloid compounds and the antifungal references such as Nystatin and Clotrimazole. Antioxidant capacity noticed that the reduction capacity of DPPH radicals obtained the best result comparatively to the others methods of free radical scavenging. Our results showed a low immunostimulatory effect and this result could be explained by the lack of biologically active antioxidants such as polyphenol compounds lowly contained in the alkaloid compounds.

**Conclusion:**

The results of this study showed that alkaloid compounds in combination with antifungal references (Nystatin and Clotrimazole) exhibited antimicrobial effects against candida strains tested. The results supported the utilization of these plants in infectious diseases particularly in treatment of candida infections.

## Background

Diseases due to pathogenic fungi represent a critical problem to human health and they are one of the main causes of morbidity and mortality worldwide [1]. The emergence of pathogens resistant to antibiotics as a result of their excessive use in clinical and veterinary applications represents a serious public health concern [2]. In the last three decades, pathogenic resistant fungi particularly candida strains, have caused major health problems throughout the world in women although the pharmacological industries produced quantities of antibiotics. Unfortunately, the resistance of fungi to these drugs is increasingly important. The search for plants with antifungal activity has gained increasing importance in recent years due to the development of resistance. On the other hand, it is known that free radicals play a fundamental role in several diseases. The biochemical damages caused by free radicals to cells and tissues, lead to the development of diseases such as arteriosclerosis, high blood pressure, cancer, inflammation, renal failure, liver disease [3].

Medicinal plants are largely used either for the prevention, or for the curative treatment of several diseases. Among the properties behind these virtues, the antioxidant activity holds the first place [4-6]. Plants used in traditional medicine may constitute an important source of new biologically active compounds. It is estimated that there are about 2,500000 species of higher plants and the majority of these plants have not been studies for their pharmacological activities [7]. According to world Health Organization (WHO), 80% of the world population still relies mainly on substances extract from plants to cure diseases [8]. The fact that microorganisms nowadays tend to develop resistance towards drugs, coupled to the undesirable side effects of certain antibiotics offer considerable potential for the development of new effective antimicrobial and antioxidant agents, medicinal plants are a prolific source. Scientific experiments on the antimicrobial properties of plant components were first documented in the late 19^th^ century [9]. The shortcomings of the drugs available today propel the discovery of new pharmacotherapeutic agents in herbal medicine [10]. Amongst the medicinal plants investigated in our research team, the family Malvaceae is largely represented and some of these plants as *Sida cordifolia* L. (Malvaceae). A decoction of this Malvaceae is locally used in the treatment of coughs, rheumatic and abdominal pain, and diarrhea while the leaf decoction is used in the treatment of fever and to prevent miscarriage [11].

Some biological activities of these Malvaceae have been reported [12]. However, to the best of our knowledge, no information of alkaloid fractions on its antioxidant, antifungal activities and immunostimulatory capacity are available.

Many fungi are parasite on plants and animals (including humans) causing serious diseases in human such as candidose among others. Furthermore, persons with immune-deficiencies are particularly susceptible to diseases by candida [13]. Supported especially a reduction in the immune system due to a lack of antioxidants, this study was therefore designed to evaluate the possible beneficial antioxidant, anticandicidal and immunostimulatory potencies of the total alkaloid fractions from *Sida cordifolia* L. (Malvaceae). Our results would strengthen the traditional use of this Malvaceae and contribute to the acceptance of traditional medicine for the best management of candidoses of which the women suffer much nowadays.

## Materials

### Plant materials

*Sida cordifolia* L., was collected in August 2008 in Gampela, 25 Km east of Ouagadougou, capital of Burkina Faso. The plant was botanically identified by Prof. Millogo-Rasolodimby from the plants Biology Department of the University of Ouagadougou. Voucher specimen was deposited in the Herbarium of the La.B.E.V. (Laboratory of Plant Ecology and Biology, UFR/SVT of University of Ouagadougou) from the University of Ouagadougou.

### Animals handling

Swiss NMRI mice (25–30 g) and adult albinos Wistar rats (195–200 g) of both sexes were used for this study. All animals were housed in cages under controlled conditions of 12-h light/and 12 h without light and 25°C. They received pellets of food enriched with 20% protein and water ad libitum. They were deprived of food for 15 h (but with access to drinking water) and weighed before the experiments. Experiments on the animals were performed according to the protocols already approved by the Institute of Health Sciences Research/University of Ouagadougou (Burkina Faso) and met the international standards for animal study [14].

### Test organisms

The studies microorganisms included reference strains of *Candida albicans* ATCC 9002, *Candida albicans* ATCC 2091, *Candida parapsilosis* ATCC 22019, *Candida tropicalis* ATCC 750, *Candida Krusei* ATCC 6258. Fungal strains were maintained on agar slant at 4°C and sub-cultured on a fresh appropriate agar plates 24 h prior to any antifungal activity. Sabouraud Glucose Agar was used for the activation of fungi. The Mueller Hinton Broth (MHB) was used for the MIC and MFC determinations.

## Methods

### Preparation of extract for acute toxicity study

Fifty grams of powdered plant materials (dried in laboratory condition) was extracted with 500 ml of acetone 80% (400 ml acetone + 100 ml water) for 24 h under mechanic agitation (SM 25 shaker, Edmund BÜHLER, Germany) at room temperature. After filtration, acetone was removed under reduced pressure in a rotary evaporator (BÜCHI, Rotavopor R-200, Switzeland) at approximately 40°C and freeze-dried (Telstar Cryodos 50 freeze-dryer). The extract was weighed before packing in waterproof plastic flasks and stored at 4°C until use.

### Extraction of alkaloid compounds

The harvested plant materials fresh (broken into leaf stems) were dried in the laboratory at room temperature (20-25°C), afterwards samples were ground and made alkaline and 50 g were used with 28% ammonia and extracted with chloroform at room temperature for a total period of 24 h and then the extract was partitioned between 5% HCl and Chloroform. The aqueous phase was made alkaline again with ammonia and partitioned between water and chloroform. Finally chloroform was totally evaporated from the organic phase to form the alkaloids powder [15].

### Antioxidant activity determination

#### DPPH radical method

Radical scavenging activity of plant fractions against stable DPPH (2, 2’-diphenyl-1-picrylhydrazyl, Fluka) was determined with a UV/visible light spectrophotometer (CECIL CE 2041, CECIL Instruments, England) at 517 nm as described by [16]. Fraction solutions were prepared by dissolving 10 mg of dry extract in 10 ml of methanol. The samples were homogenized in an ultrasonic bath. 0.5 ml of aliquots which were prepared at different concentrations from each sample of fraction was mixed with 1 ml of methanolic DPPH solution (20 mg/ml). After 15 min in the dark at room temperature, the decrease in absorption was measured. All experiments were performed in triplicate and expressed in mmol Ascorbic Acid Equivalent per g of fraction (Y= −16.815x+6.8373; R^2^ =0.9976). Quercetin was used as positive control [17,18].

### ABTS radical cation decolorization assay

For ABTS radical cation decolorization assay, the procedure followed the method of [16]. ABTS was dissolved in water to a 7 mM concentration. ABTS radical cation (ABTS^·+^) was produced by reacting ABTS stock solution with 2.45 mM potassium persulfate (final concentration) and allowing the mixture to stand in the dark at room temperature for 12 h before use. This mixture was diluted with ethanol to give an absorbency of 0.7 ± 0.02 units at 734 nm using a UV/visible light spectrophotometer (CECIL CE 2041, CECIL Instruments, England). For our study, we used 10 μL of the diluted sample (1 mgmL^-1^ in methanol) which was allowed to react with 990 μL of fresh ABTS^·+^ solution and the absorbance was taken 6 min exactly after initial mixing. Ascorbic acid was used as standard (Y= −0.0342x+0.634; R^2^ = 0.9996) and the capacity of free radical scavenging was expressed as mmol Ascorbic Acid Equivalent per g of fraction. Quercetin, a reference compound was used as positive control.

### Fe ^3+^ to Fe ^2+^ reduction activity (FRAP)

The FRAP assay was performed according to [19]. 0.5 mL of each fraction (1 mgmL-1) was mixed with 1.25 mL of phosphate buffer (0.2M, pH 6.6) and 1.25 mL of aqueous potassium hexacyanoferrate [K_3_Fe(CN)_6_ solution (1%). After 30 min incubation at 50°C, 1.25 mL of trichloroacetic acid (10%) was added and the mixture was centrifuged at 2000 × g for 10 min. Then, the upper layer solution (0.625 mL) was mixed with distilled water (0.625mL) and a freshly prepared FeCl_3_ solution (0.125mL, 0.1%). Absorbencies were read at 700 nm on a UV/visible light spectrophotometer (CECIL CE 2041, CECIL Instruments, England) and Ascorbic acid was used to produce the calibration curve (Y= 0.008×-0.0081; R^2^ = 0.9999). The iron (Fe^3+^) reducing activity determination was performed in triplicate and expressed in mmol Ascorbic Acid Equivalent per g of fraction. Troloc, a reference compound was used as positive control [20].

### *In vitro* antifungal activity

#### Preparation of inocula

The fungal strains grown on nutrient agar (Muller Hinton broth) at 35°C for 72 h were suspended in a saline solution (0.9%, w/v) NaCl and adjusted to a turbidity of 0.5 Mac Farland standard **(**5×10^5^ CFU/ml) [21].

#### Preparation of fraction substances

The stock solutions of AC were dissolved in 10% dimethylsulfoxide (DMSO) in water [21,22] at a final concentration of 800 μg/ml. The stock solutions were sterilized by filtration through 0.22 μm sterilizing Millipore express filter.

### Minimum inhibitory concentration (MIC) assay

Minimum inhibitory concentration (MIC) was determined by the microdilution method in culture broth as recommended by [21,23] with low modifications. 12 serial two-fold dilutions of AC solutions or conventional antibiotic were prepared as described before, to obtain final concentration ranges of 800–0.78125 μg/ml and 50–0.0488 μg/ml for AC and reference substances respectively. The last wells (n°12) served as sterility controls (contained broth only) or negative control (broth + inoculums). The 96-well micro-plates (NUNC, Danemark) containing 100 μL of Mueller Hinton (MH) broth were used. For each fungi strain, three columns of eleven wells to the micro-plate were used. Each well has getting: the culture medium + AC solution or Nystatin/Clotrimazole or the combination of fraction solution with Nystatin/Clotrimazole + inoculum standardized at 5×10^5^ CFU/ml (10 μl of inocula) and INT (50 μl; 0.2 mg/ml for 30 min). The plates were sealed with parafilm, then agitated with a plate shaker to mix their contents and incubated at 35°C for 48 h. All tests were performed in triplicate and the fungi activity was expressed as the mean of inhibitions produced. Viable microorganisms reduced the yellow dye to a pink colour. The MIC was defined as the lowest concentration of AC substance at which no colony was observed after incubation. So, the MIC was defined as the lowest concentration where no change was observed, indicating no growth of microorganism.

### Minimum fungicidal concentration (MFC)

Minimum fungicidal concentration (MFC) was determined by the microdilution method in culture broth as recommended by [21,23] with low modifications. Minimum fungicidal concentration (MFC) was determined by adding 50 μl aliquots of the clear wells to 150 μl of freshly prepared broth medium and incubating at 35°C for 48 h. The MFC was regarded as the lowest concentration of test sample which did not produced a colour. All tests were performed in triplicates.

### Evaluation of the fractional inhibitory concentration index of AC

The Muller Hinton agar dilution method was used to evaluate the Fractional Inhibitory Concentration Index (FICI) of AC and the tested anti-microbial standard as reported earlier [24,25]. Eleven (11) serial two-fold dilutions of AC solutions were prepared as described before, to obtain final concentration range of 800 to 0.78125 μg/ml. A series of two-fold serial dilutions of Nystatin or Clotrimazole was also prepared in the same conditions as AC. In this way, antifungal standard dilutions were mixed with the appropriate concentration of AC solution thus obtaining a series of the combinations of conventional antifungal and AC solution. The concentrations prepared corresponded to 1-1/1024 of MIC values. The 96-well micro-plate (NUNC, Danemark) containing 100 μL of Mueller Hinton (MH) broth were used. For each fungal strain, three columns of eleven wells to the micro-plate were used. Each well has getting: the culture medium + combination of AC solution with Nystatin/Clotrimazole + inoculum standardized at 5×10^5^ CFU/ml (10 μl of inocula) and INT (50 μl; 0.2 mg/ml for 30 min). The plates were covered and incubated at 35°C for 48 h. All tests were performed in triplicate and the fungicidal activity was expressed as the mean of inhibitions produced. Viable microorganisms reduced the yellow dye to a pink colour. The analysis of the combination of AC solution and antifungal references (Nystatin and Clotrimazole) was obtained by calculating the Fractional Inhibitory Concentration Index (FICI) as follows: FICI= (MICa of AC in combination/MICa alone) + (MICb of the standard antifungal agent in combination/MICb alone), where MICa (Minimal Inhibitory Concentration of AC) and MICb (Minimal Inhibitory Concentration of Nystatin or Clotrimazole). The FICI was interpreted as follows: (1) a synergistic effect when FICI =0.5; (2) an additive or indifferent effect when FICI >0.5 and <3C; 1; (3) an antagonistic effect when >1 [21].

### Immunostimulatory potential of AC on response immune specific

#### Acute toxicity study in mice

Healthy male and female Swiss mice (20-30g) were randomly divided into 7 groups (1 control group and 6 treated assay groups) of 6 animals (3 male and 3 female). The animals were deprived of food but not water 15 h prior to the administration of the test suspension. The control group received intraperitoneally. 10% dimethylsulfoxide (DMSO) solution (in water). The general behaviour of the mice was observed at 120 min after the treatment. The animals were fed with food pellets and water *ad libitum*. They were screened for morbidity and mortality once a day for up 14 days. The number of survivors after the 14 days period was noted. The toxicological effect was assessed on the basis of mortality, which was expressed as the median lethal dose (LD_50_). The LD_50_ (Lethal Dose 50 was determined according to the method of [26].

### Animals’ treatment for immunostimulatory potential

The animals were divided into 06 groups of six animals each one. Groups 1, 2 and 3 were used as controls groups.

Group 1. The rats received 10% DMSO as control by oral way during 28 days.

Group 2. The rats received cyclosporin A as control (25 mg/kg, by oral route) during 07 days (from 1^st^ to 7^th^ day) and then received DMSO 10% (from 8^th^ to 28^th^ day).

Group 3. The rats received concavanalin A (25 mg/kg, by oral route) from 8^th^ to 28^th^ day

Group 4. The rats received extract of AC from *Sida cordifolia* (50 mg/kg bw.)

Group 5. the rats received extract of AC from *Sida cordifolia* (100 mg/kg bw.)

Group 6. The rats received extract of AC from *Sida cordifolia* (200 mg/kg bw.)

The animals of groups 2 to 6 initially received cyclosporin A (25 mg/kg bw oral route) 1^st^ to 7^th^ day in order to lower the immune system. The 28^th^ day, the animals were deprived of water and food during 15 hours [27].

### Assessment of immunostimulatory potential

At the end of 28-days period, the animals were deprived of food for 15 h and blood samples were collected by cardiac puncture in two tubes for hematological and serologic parameters analysis. The blood samples (with heparin and without anticoagulant) were centrifuged at 3000 rpm for 5 min to obtain plasma or serum. Hematological analyses were performed on whole blood, using automatic counter (Mindray Auto hematology Analyser BC-5500) to evaluate following parameters: total white blood cells (TWBC), total lymphocytes using automatic Counter System (SB FACS) serologic parameters (CD8 and CD4) were determined [27].

### Statistical analysis

The data of antioxidant and antifungal activities were expressed as Mean±Standard deviation (SD) of three determinations. Statistical analysis (ANOVA with a statistical significance level set at p<3C;0.05 and linear regression) was carried out with XLSTAT 7.1. However, the data of immunostimulatory effect were expressed as Mean ± Standard deviation (SD) of six determinations (n=6). Results were analysed by one-way ANOVA followed by Dunnett’s *t*-test using Prism 4 software. The level of significance was accepted at p≤0.05.

## Results

### Free radical scavenging capacity

#### DPPH radical method

Results are consigned in the (Table 1). The reduction capacity of DPPH radicals was determined by the decrease of the absorbance induced by antioxidant at 517 nm, which is induced by antioxidant. The value is 6.63±0.10 mmoL AAE/g fractions*.* Control compound gave 13.76±0.26 mmoL AAE/g fraction for Quercetin. The different letters in the same column of Table 1 indicate significant difference (P<3C;0.05) for our different fractions.

**Table 1:** **Antioxidant properties of AC from*Sida cordifolia***

**Fractions**	**DPPH mmoL AAE/g fraction**	**FRAP mmoL/g fraction**	**ABTS mmoL AAE/g fraction**
**AC/*Sida cordifolia***	6.63±0.10	3.47±0.001	3.90±0.03
**Quercetin**	13.76±0.26	Not determined	7.81±0.21
**Trolox**	Not determined	7.46±3.38	Not determined

mmoL AAE/g fraction: mmol equivalent Ascorbic Acid for 1g dried fraction.

Values are Mean ±SD (n=3). Different letters in the same column indicate significant difference (P<3C;0.05) for AC.

### Fe ^3+^ to Fe ^2+^ reduction activity (FRAP)

For FRAP assay, the following value is 3.47±0.01 mmoL AAE/g fractions. Control compound gave 7.46±3.38 mmoL AAE/g fractions for Trolox (Table 1). The different letters in the same column of Table 1 indicate significant difference (P<3C;0.05) for our different fractions.

### ABTS radical cation decolorization assay

For ABTS radical cation decolorization assay, the value is 3.90±0.03 mmoL AAE/g fractions. The reference compound is Quercetin 7.81±0.21 mmol AAE/g fraction. We note that, the reduction capacity of DPPH radicals obtained the best result comparatively to the others methods of free radical scavenging; but comparatively to the reference compounds used we notice that the result is not interesting (Table 1). The different letters in the same column of Table 1 indicate significant difference (P<3C;0.05) for our different fractions.

### *In vitro* antifungal activity

#### Minimum inhibitory concentration (MIC) assay and Minimum fungicidal concentration (MFC)

As for the Minimum inhibitory concentration assay (MIC) and Minimum fungicidal concentration (MFC) of AC and their combination with antifungal references (Nystatin and Clotrimazole), result varied according to microorganism and results are summarize in Tables 2, 3 and 4. The MIC values of AC were ranged from 8.33 to 12.5 μg/ml.

**Table 2:** **Minimal Inhibitory Concentration (MIC) of AC from*Sida cordifolia*L., and antifungal references (Nystatin and Clotrimazole)**

**Microorganisms**	**MIC (μg/ml) AC of*Sida cordifolia***	**MIC (μg/ml) Nystatin**	**MIC(μg/ml) Clotrimazole**
***Candida albicans*ATCC 9002**	10.42±3.61^b^	4.17±1.80	4.17±1.80
***Candida albicans*ATCC 2091**	8.33±3.61^b^	4.17±1.80	6.25±0.00
***Candida parapsilosis*ATCC 22019**	12.5±0.00^a^	6.25±0.00	6.25±0.00
***Candida krusei*ATCC 6258**	8.33±3.61^c^	4.17±1.80	4.17±1.80
***Candida tropicalis*ATCC 750**	10.42±3.61^b^	4.17±1.80	6.25±0.00

Values are Mean ±SD (n=3). Different letters in the same column indicate significant difference (P<3C;0.05) for AC.

**Table 3:** **Minimal Fungicidal Concentration (MFC) of AC from*Sida cordifolia*L., and antifungal references (Nystatin and Clotrimazole)**

**Microorganisms**	**MFC (μg/ml)**	**MFC (μg/ml)**	**MFC (μg/ml)**
	**AC of*Sida cordifolia***	**Nystatin**	**Clotrimazole**
***Candida albicans*ATCC 9002**	33.33±14.43^b^	16.67±7.22	14.58±9.55
***Candida albicans*ATCC 2091**	29.17±19.09^c^	12.5±0.00	12.5±0.00
***Candida parapsilosis*ATCC 22019**	41.67±14.43^a^	14.58±9.55	16.67±7.22
***Candida krusei*ATCC 6258**	29.17±19.09^c^	12.5±0.00	14.58±9.55
***Candida tropicalis*ATCC 750**	33.33±14.43^b^	14.58±9.55	12.5±0.00

Values are Mean ±SD (n=3). Different letters in the same column indicate significant difference (P<3C;0.05) for AC.

**Table 4:** **Minimal Inhibitory Concentration (MIC) of combination of AC from*Sida cordifolia*L. and antifungal references (Nystatin and Clotrimazole)**

**Microorganisms**	**MIC (μg/ml) combination of AC + Nystatin**	**MIC (μg/ml) combination of AC + Clotrimazole**
***Candida albicans*ATCC 9002**	1.30±0.45^a^	1.30±0.45^b^
***Candida albicans*ATCC 2091**	1.04±0.45^b^	1.30±0.45^b^
***C. parapsilosis*ATCC 22019**	1.30±0.45^a^	1.56±0.00^a^
***C. krusei*ATCC 6258**	1.04±0.45^b^	1.30±0.45^b^
***C. tropicalis*ATCC 750**	1.04±0.45^b^	1.56±0.00^a^

Values are Mean ±SD (n=3). Different letters in the same column indicate significant difference (P<3C;0.05) for AC.

### Evaluation of the fractional inhibitory concentration index of fractions

For FICI, our results indicate a synergistic effect between AC and the antifungal references (Nystatin and Clotrimazole) (Table 5 and Table 6).

**Table 5:** **Fractional Inhibitory Concentration (FIC) and FICI of combination of AC from*Sida cordifolia*L. with Nystatin**

**Microorganisms**	**FIC**_**a**_	**FIC**_**b**_	**FICI**	**Effect**
***C. albicans*ATCC 9002**	0.12	0.31	0.43	Synergistic
***C. albicans*ATCC 2091**	0.12	0.25	0.37	Synergistic
***C. parapsilosis *ATCC 22019**	0.10	0.21	0.31	Synergistic
***C. krusei*ATCC 6258**	0.12	0.25	0.37	Synergistic
***C. tropicalis*ATCC 750**	0.10	0.25	0.35	Synergistic

**FIC**_**a**_ = MIC of AC in combination/MICa alone; **FIC**_**b**_ = MIC of the antifungal agent in combination/ MICb alone and FICI= FIC_a_ + FIC_b_.

a= alkaloid compounds; b= Nystatin.

The FICI was interpreted as follows: (1) a synergistic effect when FICI =0.5; (2) an additive or indifferent effect when FICI >0.5 and <3C;1; (3) an antagonistic effect when >1.

**Table 6:** **Fractional inhibitory concentration (FIC) and FICI of combination of n-butanol fraction from*Sida cordifolia*L. with Clotrimazole**

**Microorganisms**	**FIC**_**a**_	**FIC**_**b**_	**FICI**	**Effect**
***C. albicans*ATCC 9002**	0.12	0.31	0.43	Synergistic
***C. albicans*ATCC 2091**	0.12	0.21	0.33	Synergistic
***C. parapsilosis*ATCC 22019**	0.10	0.25	0.35	Synergistic
***C. krusei*ATCC 6258**	0.12	0.31	0.43	Synergistic
***C. tropicalis*ATCC 750**	0.10	0.25	0.35	Synergistic

**FIC**_**a**_ = MICa alone /MIC of AC in combination; **FIC**_**b**_ = MICb of standard antifungal agent/MIC of the antifungal agent in combination and FICI= FIC_a_ + FIC_b_.

a= alkaloid compounds; b= Clotrimazole.

The FICI was interpreted as follows: (1) a synergistic effect when FICI =0.5; (2) an additive or indifferent effect when FICI >0.5 and <3C;1; (3) an antagonistic effect when >1.

### Acute toxicity study in mice

About the acute toxicity study in mice, the value of LD50 was: 3.4 g/kg. The results of this study indicated that the extract of *Sida cordifolia* are low poisonous. During the 14 day period of acute toxicity evaluation, some signs of toxicity were observed, but they were all quickly reversible.

### Immunostimulatory potential of AC on response immune specific

**Figure 1:**
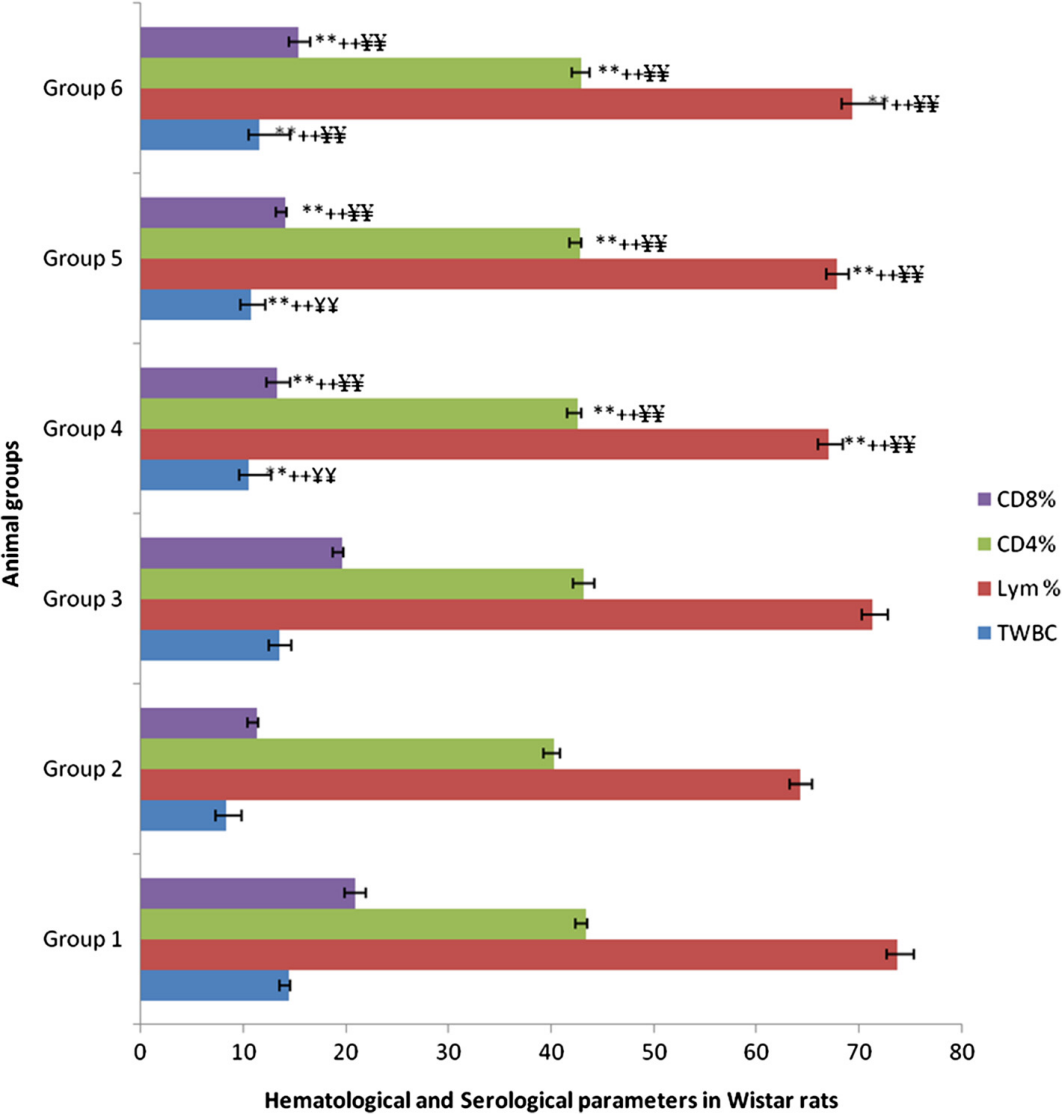
**Effects of AC from *Sida cordifolia *L. on hematological and serological parameters in wistar rats.** TWBC (10^3^/μl); Lym= Total Lymphocytes. Values are mean ± S.E.M. (n=6) one-way ANOVA followed by Dunnett’s *t*- test: Compare all vs. control: **++ ¥¥ p<3C;0.01 compared with control groups (DMSO 10%, Cyclosporin A and Concavanalin A). Group 1: rats received 10% DMSO during 28 days. Group 2: rats received cyclosporin A (25 mg/kg bw.) from 1^st^ to the 7^th^ day and 10% DMSO from 8^th^ to the 28^th^ day. Group 3: rats received cyclosporin A (25 mg/kg bw.) from 1^st^ to the 7^th^ day and received Concavanalin A (25 mg/kg bw.) dissolved in 10% DMSO from the 8^th^ day to the 28^th^ day. Group 4: rats received cyclosporin A (25 mg/kg bw.) from 1^st^ to the 7^th^ day and received AC from *Sida cordifolia* (50 mg/kg bw.) dissolved in 10% DMSO from the 8^th^ day to the 28^th^ day. Group 5: rats received cyclosporin A (25 mg/kg bw.) from 1^st^ to the 7^th^ day and received AC from *Sida cordifolia* (100 mg/kg bw.) dissolved in 10% DMSO from the 8^th^ day to the 28^th^ day. Group 6: rats received cyclosporin A (25 mg/kg bw.) from 1^st^ to the 7^th^ day and received AC from *Sida cordifolia* (200 mg/kg bw.) dissolved in 10% DMSO from the 8^th^ day to the 28^th^ day.

At last, concerning the effect of alkaloid fractions on specific immune response, Figure 1 shows the effects of alkaloid compounds on the hematologic and serologic parameters of the rats. It is noticeable that the effect of the total alkaloid fractions concentrations of 50 mg/kg, 100 mg/kg and 200 mg/kg bw involved a significant decrease of hematologic and serologic parameters (p<3C;0.01) compared to the group controls. On hematological and serological parameters (Figure 1), there is actual evidence that cyclosporin A has an immunosuppressive potential and concavanalin A has an immunostimulatory property. The low increase in hematological and serological parameters of the test group 6, compared to the control group 2 (cyclosporin A control) allows to say that the total alkaloid fractions (200 mg/kg bw; p<3C;0.01) has a low immunostimulatory effect.

## Discussion

Infectious diseases caused by fungi are still a major threat to public health, despite numerous efforts by researchers. Their impact is particularly large in developing countries due to the relative unavailability of medicines and the emergence of widespread drug resistance [28]. Use of ethnopharmacological knowledge is one attractive way to reduce empiricism and enhance the probability of success in new drug-finding efforts [29]. Validation and selection of primary screening assays are pivotal to guarantee sound selection of extracts or molecules with relevant pharmacological action and worthy following up [30]. The number of multi-drug resistant microbial strains and the appearance of strains with reduced susceptibility to antibiotics are continuously increasing. This increase has been attributed to indiscriminate use of broad-spectrum, immunosuppressive agent and ongoing epidemics of HIV infection [31]. The development of drug resistance in human pathogens against commonly used antibiotics and antifungal has necessitated a search for new antimicrobial substances. Higher plants produce diverse secondary metabolites with different biological activities. These natural compounds may be a source of compounds with antimicrobial effects and therefore possible candidates for the development of new antifungal agents [32,33].

On the toxicity of the extract, according to [34], pharmacological substances whole LD_**50**_ is less than 5 mg/kg body weight are classified in the range of highly toxic substances, those with a LD_**50**_ between 5 mg/kg body weight and 5000 mg/kg body weight are classified in the range of moderately toxic substances and those with the lethal dose is more than 5000 mg/kg body weight not toxic. In this fact, if we refer to this classification we could say that the extract of *Sida cordifolia* is moderately toxic and would be regarded as being safe or of low toxicity [35].

For the antioxidants properties, we have tested three methods for a best appreciation of our results; because a recent study demonstrates that there are differences between the test systems for the determination of the antioxidants properties [36]. The phytochemical analysis carried out on *Sida cordifolia* L., extract show that several polyphenol including flavonoids are found in its extracts [11]. Generally, the chemical structure of flavan-3-ol family has good antioxidants response towards DPPH^*^ radical. The hydrogen donating substituents (hydroxyl groups), attached to aromatic ring structures of flavonoids, which enable the flavonoids to undergo a redox reaction that helps them to scavenge the free radicals [37]. So, it can be observed that the content of polyphenolic compounds of *Sida cordifolia* could be responsible for the radical scavenging activity. Thus, the restoration of the antioxidant defense system in this study may be due in part to the antioxidant activity of polyphenolic compounds [38]. In effect, the amount of DPPH scavenging activity in the majority of the plant extracts is attributed to the phenolic fraction [39]. In the present study, the scavenging of the DPPH radical may be attributed to its hydrogen-donating ability. Earlier workers have observed a direct correlation between antioxidant activity and the reducing power of certain plant extracts [40]. Certainly, the reducing property is generally associated with the presence of reductones, which exert antioxidant action by breaking the free radical chain by donating a hydrogen atom [40].

The results obtained in this study about antifungal activity indicate a considerable difference in antifungal activity of alkaloid compounds and in combination with antifungal references. About these findings, it is widely known that plants possess healing properties [41]. The best of our knowledge, this is the first report dealing with the interaction between alkaloid compounds with the chemical antimicrobial drugs currently in use. Synergy research in phytomedicine has established as a new key activity in recent years. It is one main aim of this research to find a scientific rational for the therapeutic superiority of herbal drugs derived from traditional medicine as compared with single constituents thereof. Synergy effects of the mixture of bioactive constituents and their byproducts contained in plant extracts are claimed to be responsible for the improved effectiveness of many extracts and conventional antimicrobial drugs [42]. Such properties can be partly attributed to the diverse array of secondary metabolite such as alkaloids which are known to be essential for plants’ defense against microbial attack or insect and animal predation [43]. Moreover, it is important to notice that over compounds such as alkaloids have strong antimicrobial activities [15]. Alkaloid compounds were very susceptible fungus probably because they act on mucopolysacharide capsule of fungal strains. In effect, the polysaccharide capsular material in some of the pathogenic microorganism is responsible for virulence and antimicrobial resistance [44]. It is the reason of difference in antifungal activity of alkaloid compounds and their combination with antifungal references. Recent studies showed that the alkaloids are known to have antimicrobial properties and [45] have reported about 300 alkaloids showing such activity.

As for the immunostimulatory capacity, results could be explained by the lack of biologically active antioxidants such as polyphenol compounds lowly contained in the alkaloid compounds. In effect, polyphenol compounds are considered as the major contributors to the antioxidant potential of plants [46]*,* and antioxidant play an important role in controlling oxidative stress and decreasing disease activity [47]. This relation could be responsible for reducing oxidative stress due to cyclosporin A. These biological antioxidants by inhibiting the action of cyclosporin A have an inhibitory effect on the highly specific T helper response, eliminating the primary cell response to antigen. Indeed, after binding to an intra-cytoplasmic cyclophilin receptor, it blocks the activation pathway and calcineurin-dependent transcription and consequently gene expression of cytokines required for immune response [48]. In this case, the low immunostimulatory effect observed is due by the action of alkaloid compounds which are not the bioactive antioxidant compounds being able inhibit the oxidative stress. Indeed, surely this low immune power could be also explained by the low bioavailability of the extracts administered orally. It is worth noting that the intestinal absorption of plant extracts through the intestinal absorption is often low and weak [49].

## Conclusion

In conclusion, the screening of antioxidant, antifungal and immunostimulatory activities performed on alkaloid compounds from *Sida cordifolia* L. which was traditionally used as herbs shows that they are endowed with potentially exploitable antifungal activity. Further purification of the active compounds and their anticandicidal activity evaluation are therefore suggested for further studies.

## Competing interests

The authors declare that they have no competing interests.

## Authors’ contributions

KK and MO carried out the study and wrote the manuscript, ANL and AS supervised the work and the manuscript. BMB and LLS contributed to the manuscript corrections. All authors read and approved the final manuscript.
